# Quantitative Imaging of Pyruvate Metabolism in a Patient With Anaplastic Thyroid Cancer

**DOI:** 10.1002/mrm.70351

**Published:** 2026-03-24

**Authors:** James A. Bankson, Joshua S. Niedzielski, Collin J. Harlan, Yunyun Chen, Christopher M. Walker, Philip L. Lorenzi, Lin Tan, Vikas Kundra, Matthew E. Merritt, Mukundan Ragavan, Dawid Schellingerhout, Renjie He, Yao Ding, Sahana Datar, Qing Wang, Christine B. Peterson, Clifton D. Fuller, Vlad C. Sandulache, Stephen Y. Lai

**Affiliations:** ^1^ Department of Imaging Physics The University of Texas MD Anderson Cancer Center Houston Texas USA; ^2^ The University of Texas MD Anderson Cancer Center UTHealth Houston Graduate School of Biomedical Sciences Houston Texas USA; ^3^ Department of Radiation Physics The University of Texas MD Anderson Cancer Center Houston Texas USA; ^4^ Department of Head and Neck Surgery The University of Texas MD Anderson Cancer Center Houston Texas USA; ^5^ Metabolomics Core Facility, Department of Bioinformatics and Computational Biology The University of Texas MD Anderson Cancer Center Houston Texas USA; ^6^ Department of Abdominal Imaging The University of Texas MD Anderson Cancer Center Houston Texas USA; ^7^ Department of Biochemistry and Molecular Biology University of Florida Gainesville Florida USA; ^8^ Department of Neuroradiology The University of Texas MD Anderson Cancer Center Houston Texas USA; ^9^ Department of Radiation Oncology The University of Texas MD Anderson Cancer Center Houston Texas USA; ^10^ Department of Biostatistics The University of Texas MD Anderson Cancer Center Houston Texas USA; ^11^ Bobby R. Alford Department of Otolaryngology‐Head and Neck Surgery Baylor College of Medicine Houston Texas USA; ^12^ Department of Molecular and Cellular Oncology The University of Texas MD Anderson Cancer Center Houston Texas USA

**Keywords:** cancer metabolism, hyperpolarized pyruvate, pharmacokinetic analysis, quantitative imaging

## Abstract

**Purpose:**

Magnetic resonance imaging using hyperpolarized (HP) [1‐^13^C]‐pyruvate enables assessment of pyruvate metabolism in vivo and offers new insight into metabolic changes in response to cancer therapy. Widely used semi‐quantitative metrics of pyruvate metabolism can be affected by physiological factors that are extrinsic to intracellular metabolism. A validated pharmacokinetic (PK) model for analysis of intracellular pyruvate metabolism is needed to enhance the accuracy of quantitative metrics and clinical translation of metabolic MRI using HP pyruvate.

**Methods:**

A PK model with two physical compartments and two chemical pools was developed to analyze the conversion of labeled pyruvate into lactate in vitro. Cells exposed to [U‐^13^C_3_]‐pyruvate were analyzed using pseudo‐dynamic ion‐coupled mass spectrometry (IC‐MS) while cells exposed to HP [1‐^13^C]‐pyruvate were analyzed using dynamic NMR. The model was extended to incorporate a third physical compartment for vascular delivery, and quantification of changes in pyruvate metabolism in a patient with ATC was compared against semi‐quantitative metrics.

**Results:**

Good correspondence between complementary quantitative measures of pyruvate metabolism using IC‐MS and NMR support the use of this framework as a foundation for quantitative analysis of HP pyruvate metabolism in vitro and in vivo. The three‐compartment model identified changes in vascular delivery separately from changes in intracellular pyruvate metabolism, revealed greater heterogeneity in metabolic activity, and identified areas of persistent high metabolic activity against an overall reduction in tumor metabolism after 8 days of treatment.

**Conclusion:**

This framework for analysis provides a validated approach and demonstrates feasibility for quantitative evaluation of HP pyruvate metabolism in vivo.

## 1 Introduction

Dysregulated metabolism is a ubiquitous hallmark of cancer [1]. Otto Warburg first observed more than 100 years ago that cultured tumor tissues exhibited higher levels of glucose consumption and lactate production in the presence of oxygen [2, 3]. This phenomenon, frequently referred to as aerobic glycolysis or the Warburg effect, was long thought to be a consequence of mitochondrial dysfunction. Over the last two decades it has become apparent that aerobic glycolysis operates in conjunction with other metabolic pathways to support tumor cell growth and proliferation [4], and that significant metabolic heterogeneity can exist within and between tumors due to differences in genotype, tissue of origin, characteristics of the local tumor microenvironment, availability of oxygen and nutrients, and other metabolic stresses [5, 6]. Tumor metabolism evolves dynamically as disease progresses and responds to therapy.

Assessment of tumor metabolism that is both spatially and temporally heterogeneous in solid tumors is extremely challenging. Serial biopsies with sufficient sampling density to capture metabolic heterogeneity are incompatible with clinical practice, but minimally invasive metabolic imaging methods that can be repeated over time offer a promising alternative. Whereas positron emission tomography (PET), following administration of ^18^Fluorodeoxyglucose (FDG), is often used to assess glucose uptake, dynamic magnetic resonance imaging (MRI) of hyperpolarized (HP) [1‐^13^C]‐pyruvate can provide measurements of intra‐cellular carbon flux through aerobic glycolysis, and by inference reducing equivalent reserves, with unprecedented sensitivity, specificity, and spatiotemporal resolution [7, 8, 9]. This technology relies on an external polarizer system to transiently enhance the excess spin population of ^13^C‐enriched pyruvate, and thus the signal that is observable through MRI, by four to five orders of magnitude compared to thermal equilibrium. Spectroscopic imaging techniques allow dynamic imaging of the spatial and temporal fate of hyperpolarized pyruvate and its metabolites.

Imaging and quantification of HP MRI signal evolution are challenging in part because signal enhancement is nonstationary and transient. A detectable signal that is established by the external polarizer decays continuously towards thermal equilibrium due to T_1_ decay and is further depleted by excitation pulses that are necessary for imaging. After injection, HP pyruvate must cross multiple biological barriers before interacting with intracellular enzymes that mediate chemical conversion. Pyruvate is reversibly converted into lactate through the enzyme lactate dehydrogenase (LDH) and coenzyme nicotinamide adenine dinucleotide (NADH). The equilibrium velocity of this reaction depends on the intracellular concentration of LDH, NADH, pyruvate and lactate [10], and conversion of HP pyruvate into lactate also depends on the fraction of spin‐labeled molecules in intracellular pyruvate and lactate pools [11].

A variety of qualitative, semi‐quantitative, and quantitative metrics have been used to characterize signal evolution in HP MRI [12, 13]. The area‐under‐the‐curve (AUC) can easily be calculated for individual metabolites to provide a qualitative assessment of relative signal intensity. The AUC for a metabolite may be normalized to the standard deviation of noise in the background to yield metabolite‐specific signal‐to‐noise ratio (SNR) maps. Ratios of AUC measurements, such as the AUC of HP lactate (AUC_L_) normalized to the AUC of HP pyruvate (AUC_P_), or AUC_L_ normalized to total observed carbon signal (AUC_L_ + AUC_P_), also reflect semi‐quantitative measures with a meaningful scale. Pharmacokinetic (PK) models of HP pyruvate and lactate describe signal evolution as a function of model parameters that reflect tissue characteristics. PK analysis of HP MRI data has the potential for more targeted metabolic quantification [14, 15]. Unfortunately, PK models of HP pyruvate signal evolution that facilitate unbiased estimation of this exchange rate are relatively complex, with a large number of model parameters that must also be estimated [12]. The complexity of PK models can be tailored to balance accuracy and reproducibility in the context of the quality and characteristics of observed data to which the model would be applied [16].

Metrics of HP pyruvate metabolism have been compared with tissue correlates in preclinical and clinical studies. Perfusion, transport, and enzymatic activity represent the three primary biological factors that regulate HP pyruvate/lactate signal evolution in vivo. Delivery and metabolism of HP pyruvate have been shown to correlate with tissue perfusion [16, 17, 18, 19]. The conversion of pyruvate into lactate and other metabolites, characterized by metabolite ratios, precursor‐product PK models, and similar semi‐quantitative metrics, have been shown to correlate with monocarboxylate transporters [20, 21, 22, 23, 24, 25], enzymatic activity [22, 23, 24, 26, 27, 28, 29, 30], and tumor grade/aggressiveness [17, 22, 23, 25, 28, 30, 31, 32]. Varying correlation between these phenomena and semi‐quantitative metrics of HP pyruvate metabolism arises in part because these metrics are affected by all of these factors to varying degrees over time.

Quantification that can specifically account for these factors separately and specifically may improve our ability to characterize and understand disease progression and changes in response to therapy. Identification of an analysis approach with the correct level of complexity requires robust validation. Mass spectrometry is a well‐established technique for assessing mass‐labeled metabolites in biological samples and is analogous to the use of HP MRI to assess incorporation of spin‐labeled metabolites into intracellular metabolic processes [33, 34]. In this work, we describe the use of a three‐compartment PK model to quantify tumor metabolism using HP MRI in a patient with anaplastic thyroid cancer (ATC). This model is built upon a PK model of pyruvate metabolism tested using ion‐coupled mass spectrometry (IC‐MS) and nuclear magnetic resonance (NMR) to establish a PK model that describes transport and intracellular pyruvate metabolism in a closed, two‐compartment system containing only cells and media. We demonstrate linear correspondence between *k*_*PL*_, the apparent rate constant for conversion of labeled pyruvate into lactate, observed via NMR and IC‐MS. We then extend that model to include an intravascular pool and utilize the prior information contained within this model to more specifically assess intracellular pyruvate metabolism and changes that are induced early over the course of treatment.

## 2 Methods

The primary goal of this study was to validate a PK model for HP MRI that describes the exchange of labeled metabolites between intracellular and extracellular space in vitro, and extend that model to yield a rationally designed model with three physical compartments (3PC) that describes the exchange of labeled metabolites in vivo between vascular, extravascular/extracellular, and intracellular space. The secondary goal was to demonstrate the use of this model for quantitative analysis of HP pyruvate metabolism, and to compare this approach with other common metrics to identify early changes in tumor metabolism that are induced by therapy.

### 2.1 Cells

PC‐3 and LnCaP human prostate cancer cells were obtained from the American Type Culture Collection (ATCC, Rockville, MD). Hth‐83 human anaplastic thyroid cancer (ATC) cells were kindly provided by Dr. Jeffrey Myers (MD Anderson, Houston, TX). Cells were authenticated using short tandem repeat profiling in our institutional Cytogenetics and Cell Authentication Core.

### 2.2 Dynamic NMR Spectroscopy of Cells Exposed to [1‐ ^13^C]‐Pyruvate

Human Hth‐83 ATC, PC3 prostate, and LnCap prostate cancer cells were expanded and cultured in 150 mm dishes for 48 h, washed with phosphate buffer saline (PBS), and followed with trypsinization. Next, cells were suspended in 50 mL of Roswell Park Memorial Institute (RPMI)‐1640 medium (Sigma‐Aldrich, St. Louis, MO, USA) with 10% fetal bovine serum (FBS) (Sigma‐Aldrich, St. Louis, MO, USA) and centrifuged at 1200 rpm for 5 min. Prior to HP NMR, the cell pellet was resuspended in 1.25 mL RPMI‐1640 medium containing 10% FBS. Average cell diameter and total cell count were measured using a Cellometer Auto 2000 cell viability counter (Nexcelom Biosciences, Lawrence, MA, USA).

Approximately 1 × 10^7^ cells (Hth‐83 ATC; PC3 and LnCap prostate cancer) were suspended in 900 μL of cell media and added to a 10 mm diameter Shigemi tube (SP Wilmad‐LabGlass, Vineland, NJ, USA), along with 100 μL of D_2_O (Sigma‐Aldrich, St. Louis, MO, USA) [35]. Sample size was limited to ensure it would be contained within the homogeneous region of the NMR coil to minimize nonuniform B_1_ as a source of bias in PK analysis of HP signal evolution. The Shigemi tube was lowered to the isocenter of the 7 T NMR scanner, and the bore temperature was maintained at 310 K for ˜15 min to allow cells to achieve thermal equilibrium. During this time, standard NMR locking, tuning, and shimming calibrations were executed using TopSpin (Bruker, Billerica, MA, USA).

Approximately 181 μL of 20 mM HP [1‐^13^C]‐pyruvate, prepared as previously described using a HyperSense dissolution dynamic nuclear polarization system (Oxford Instruments, Abingdon, England, UK), was rapidly administered through tubing which spanned from the top of the bore to the bottom of the cell suspension in the Shigemi tube, resulting in a nominal concentration of approximately 3.07 mmol/L pyruvate [35]. Immediately afterwards, a 5 mL syringe was used to push air through the tubing to agitate cells and ensure mixture. Dynamic ^13^C spectroscopy was initialized immediately prior to injection of the HP pyruvate into the sample, using a pulse acquire sequence with a 2 s repetition time and a 10° excitation angle. Dynamic signals for HP pyruvate and lactate were obtained by calculating the area under a Lorentzian fit to each metabolite at each timepoint.

### 2.3 Ion‐Chromatography Mass Spectrometry of Cells Exposed to [U‐ ^13^C_3_ ]‐Pyruvate

**FIGURE 1:**
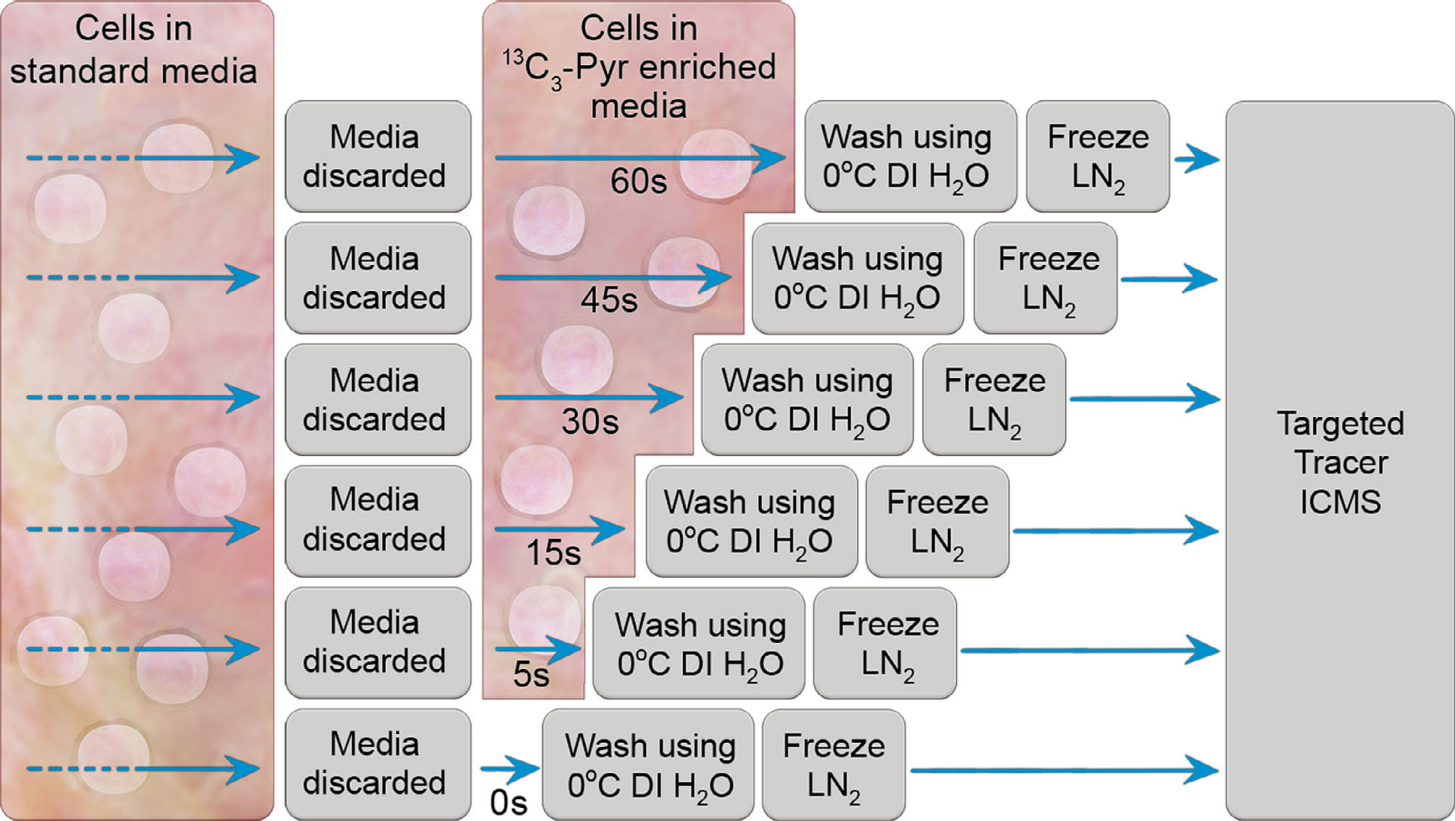
Flowchart for pseudo‐dynamic assessment of the conversion of mass‐labeled pyruvate into lactate using ion‐chromatography mass spectrometry (IC‐MS). Cells were cultured in normal media, which was discarded and immediately replaced by normal media enriched with 1 mmol/L [U‐^13^C_3_]‐pyruvate for the specified time. Enriched media were then discarded, and cells were immediately washed with ice‐cold deionized water, then with liquid nitrogen and harvested for analysis using IC‐MS.

Human Hth‐83 ATC, PC3 prostate, and LnCap prostate cancer cells were expanded and cultured in RPMI‐1640 medium (Sigma‐Aldrich, St. Louis, MO, USA), along with 10% FBS (Sigma‐Aldrich, St. Louis, MO, USA), 2 mmol/L L‐glutamine, and non‐essential amino acids (Cambrex BioScience, Walkersville, MD, USA) in a 37°C incubator supplied with 95% O_2_ and 5% CO_2_. Approximately 80% confluent cells were seeded in 60 mm dishes. An hour prior to the IC‐MS experiments, the cell media was refreshed with 5 mL cell culture medium. At the start of each experiment, the media was discarded and the cells were incubated in fresh RPMI‐1640 medium containing 1 mM [U‐^13^C_3_]‐pyruvate (Cambridge Isotope Laboratories, Tewksbury, MA, USA) for 0, 5, 15, 30, 45, and 60 s. Cells were then quickly washed with ice‐cold deionized water to halt metabolism and then frozen by the addition of approximately 5 mL of liquid nitrogen (Figure 1). Metabolites were extracted using an ice‐cold mixture of methanol and water (methanol: water = 80:20 [vol/vol]). Samples were centrifuged at 17000*g* for 5 min at 4°C, with supernatants being transferred to clean tubes and then followed by evaporation to dryness under nitrogen. Samples were reconstituted in deionized water and 5 μL was injected into a Thermo Scientific Dionex ICS‐5000 + IC system containing a Thermo IonPac AS11250 × 2 mm 4 μm column. A flow rate of 350 μL/min at 30°C was utilized. The gradient conditions were as follows: 0–25 min, 1–35 mM KOH; 25–39 min, 35–99 mM KOH; 39–49 min, 99 mM KOH; 49–50 min, 1 mM KOH. To improve desolvation and sensitivity, methanol was added post‐column by an external pump via a low dead‐volume mixing tee.

Data were acquired using a Thermo Orbitrap Fusion Tribrid Mass Spectrometer under ESI negative mode at a resolution of 240 000 (FWHM at m/z 200) for MS1 acquisition. Raw files were imported into Thermo Trace Finder software for final analysis. Relative abundance of labeled pyruvate and lactate was calculated as the area under the curve of the extracted ion chromatogram corresponding to the M + 3 isotopologs. Peak areas and fractional enrichments were corrected for natural abundance using the ElemCor software tool [36].

Aliquots of cells exposed to 1 mM [U‐13C3]‐pyruvate for 0, 5, 15, 30, 45, and 60 s and suspended in ice‐cold methanol/water were also analyzed by 1H NMR at 14.1 T using a 1.7 mm dual‐tuned high‐temperature cryoprobe (Bruker, Billerica, MA, USA). To estimate the fractional enrichment of the C3 of lactate, interleaved spectra were acquired with 13C broadband GARP decoupling either on or off.

### 2.4 Pharmacokinetic Modeling of Cells in Labeled Media

**FIGURE 2:**
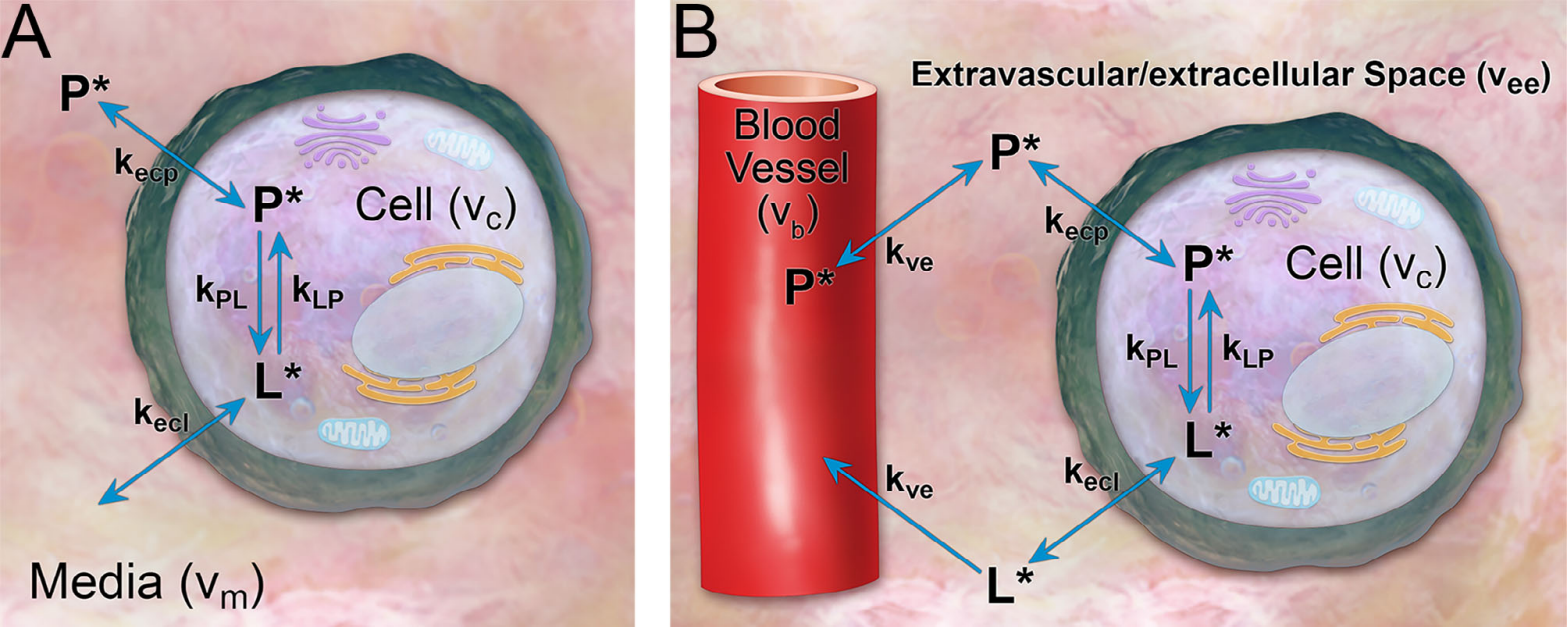
Pharmacokinetic models for pyruvate metabolism. (A) The in vitro model for cells in media is a closed system with two physical compartments and two chemical pools. Labeled pyruvate is introduced through the media and crosses into cytosol, predominantly via MCT1, at the rate indicated by *k*_*ecP*_. Labeled pyruvate is converted into lactate at a rate given by *k*_*PL*_ through interaction with intracellular enzymes lactate dehydrogenase (LDH) and nicotinamide‐adenine dinucleotide (NADH), while *k*_*LP*_ denotes the rate of the reverse reaction. Labeled lactate leaves the cell (primarily via MCT4) at the rate given by *k*_*ecL*_. The exchange of label between physical compartments and chemical pools is described mathematically by Equations ((1), (2), (3), (4), (5), (6)). (B) For analysis in vivo, the model is extended to include three physical compartments (vascular, extravascular/extracellular, and intracellular space) and two chemical pools, as described by Equations (3, 4), and ((9), (10), (11), (12)).

To characterize the dynamic incorporation of mass/spin labels into intracellular metabolic processes, a two‐compartment pharmacokinetic (PK) model was developed that includes two physical compartments and two chemical pools (Figure 2A):

(1) $$\frac{\delta {\mathrm{Pyr}}_{m}^{*}\left(t\right)}{\delta t}=-\frac{k_{\mathrm{ecP}}}{v_{m}}\left({\mathrm{Pyr}}_{m}^{*}\left(t\right)-{\mathrm{Pyr}}_{c}^{*}\left(t\right)\right)-R_{1,\mathrm{Pyr}}{\mathrm{Pyr}}_{m}^{*}\left(t\right)$$

(2) $$\frac{\delta {\mathrm{Lac}}_{m}^{*}\left(t\right)}{\delta t}=-\frac{k_{\mathrm{ecL}}}{v_{m}}\left({\mathrm{Lac}}_{m}^{*}\left(t\right)-{\mathrm{Lac}}_{c}^{*}\left(t\right)\right)-R_{1,\mathrm{Lac}}{\mathrm{Lac}}_{m}^{*}\left(t\right)$$

(3) $$\begin{array}{ll} \frac{\delta {\mathrm{Pyr}}_{c}^{*}\left(t\right)}{\delta t} & =-\frac{k_{\mathrm{ecP}}}{v_{c}}\left({\mathrm{Pyr}}_{c}^{*}\left(t\right)-{\mathrm{Pyr}}_{m}^{*}\left(t\right)\right)-\left(k_{\mathrm{PL}}+R_{1,\mathrm{Pyr}}\right){\mathrm{Pyr}}_{c}^{*}\left(t\right)\\ & \;+k_{\mathrm{LP}}{\mathrm{Lac}}_{c}^{*}\left(t\right) \end{array}$$

(4) $$\begin{array}{ll} \frac{\delta {\mathrm{Lac}}_{c}^{*}\left(t\right)}{\delta t} & =-\frac{k_{\mathrm{ecL}}}{v_{c}}\left({\mathrm{Lac}}_{c}^{*}\left(t\right)-{\mathrm{Lac}}_{m}^{*}\left(t\right)\right)-\left(k_{\mathrm{LP}}+R_{1,\mathrm{Lac}}\right){\mathrm{Lac}}_{c}^{*}\left(t\right)\\ & \;+k_{\mathrm{PL}}{\mathrm{Pyr}}_{c}^{*}\left(t\right) \end{array}$$

Labeled pyruvate and lactate, observable by mass spectrometry or magnetic resonance, are adorned with an asterisk to differentiate endogenous from exogenous, labeled molecules. Spatial compartments are identified with subscripts “c” for intracellular and “m” for extracellular/media, and their relative volume fractions sum to unity (*v*_*m*_ + *v*_*c*_ = 1). The first term on the right‐hand side of each equation above describes flux between physical compartments; pyruvate crosses from extracellular space to intracellular space according to a rate constant *k*_*ecP*_, normalized by the fractional volume of the associated spatial compartment to ensure that labels are conserved when crossing between compartments of unequal size. *R*_*1,Pyr*_ and *R*_*1,Lac*_ reflect loss of MR signal due to spin–lattice relaxation; these constants are set to zero for analysis of IC‐MS data. The rightmost term in Equations (3, 4) reflect chemical conversion of labeled molecules, which is only permitted in intracellular space. Chemical conversion is described using a first‐order, two‐site model that has been previously introduced, with apparent rate constants of *k*_*PL*_ for conversion of pyruvate to lactate, and *k*_*LP*_ for the reverse reaction.

The solution to this system of ordinary differential equations is given by:

(5) $$\left[\begin{array}{l} {\mathrm{Pyr}}_{m}^{*}\left(t\right)\\ {\mathrm{Lac}}_{m}^{*}\left(t\right)\\ {\mathrm{Pyr}}_{c}^{*}\left(t\right)\\ {\mathrm{Lac}}_{c}^{*}\left(t\right) \end{array}\right]=e^{A_{c}\left(t-t_{0}\right)}\left[\begin{array}{l} {\mathrm{Pyr}}_{m}^{*}\left(t_{0}\right)\\ {\mathrm{Lac}}_{m}^{*}\left(t_{0}\right)\\ {\mathrm{Pyr}}_{c}^{*}\left(t_{0}\right)\\ {\mathrm{Lac}}_{c}^{*}\left(t_{0}\right) \end{array}\right]$$

where

(6) $$\begin{array}{cc} & A_{c}=\\ & \left[\begin{array}{cccc} -\;\left(\frac{k_{\mathrm{ecP}}}{v_{m}}+R_{1,\mathrm{Pyr}}\right) & 0 & \frac{k_{\mathrm{ecP}}}{v_{m}} & 0\\ 0 & -\left(\frac{k_{\mathrm{ecL}}}{v_{m}}+R_{1,\mathrm{Lac}}\right) & 0 & \frac{k_{\mathrm{ecL}}}{v_{m}}\\ \frac{k_{\mathrm{ecP}}}{v_{c}} & 0 & -\left(\frac{k_{\mathrm{ecP}}}{v_{c}}+k_{\mathrm{PL}}+R_{1,\mathrm{Pyr}}\right) & k_{\mathrm{LP}}\\ 0 & \frac{k_{\mathrm{ecL}}}{v_{c}} & k_{\mathrm{PL}} & -\left(\frac{k_{\mathrm{ecL}}}{v_{c}}+k_{\mathrm{LP}}+R_{1,\mathrm{Lac}}\right) \end{array}\right] \end{array}$$

Equations (5, 6) describe the evolution of labeled metabolites in a closed system containing cells, media, and a quantity of labeled pyruvate that is added at time $t=t_{0}$ such that ${\mathrm{Pyr}}_{m}^{*}\left(t_{0}\right)=P_{0}$ at the initial exposure. Excitation losses in NMR measurements are modeled as instantaneous losses that occur at each excitation [37]. Equation (5) can be written to describe longitudinal magnetization at the *n*th excitation pulse following the previous pulse with flip angle θ:

(7) $$\left[\begin{array}{l} {\mathrm{Pyr}}_{m}^{*}\left(\mathrm{nTR}\right)\\ {\mathrm{Lac}}_{m}^{*}\left(\mathrm{nTR}\right)\\ {\mathrm{Pyr}}_{c}^{*}\left(\mathrm{nTR}\right)\\ {\mathrm{Lac}}_{c}^{*}\left(\mathrm{nTR}\right) \end{array}\right]=e^{A_{c}\cdot \mathrm{TR}}\left[\begin{array}{l} {\mathrm{Pyr}}_{m}^{*}\left(\left[n-1\right]\mathrm{TR}\right)\cos\theta \\ {\mathrm{Lac}}_{m}^{*}\left(\left[n-1\right]\mathrm{TR}\right)\cos\theta \\ {\mathrm{Pyr}}_{c}^{*}\left(\left[n-1\right]\mathrm{TR}\right)\cos\theta \\ {\mathrm{Lac}}_{c}^{*}\left(\left[n-1\right]\mathrm{TR}\right)\cos\theta \end{array}\right]$$

The observed NMR signal depends on the excitation angle of the *n*th pulse, and reflects a combination of signal from media and cells:

(8) $$\left[\begin{array}{l} {\mathrm{Pyr}}_{T}^{*}\left(\mathrm{nTR}\right)\\ {\mathrm{Lac}}_{T}^{*}\left(\mathrm{nTR}\right) \end{array}\right]=v_{m}\left[\begin{array}{l} {\mathrm{Pyr}}_{m}^{*}\left(\mathrm{nTR}\right)\sin\theta \\ {\mathrm{Lac}}_{m}^{*}\left(\mathrm{nTR}\right)\sin\theta \end{array}\right]+v_{c}\left[\begin{array}{l} {\mathrm{Pyr}}_{c}^{*}\left(\mathrm{nTR}\right)\sin\theta \\ {\mathrm{Lac}}_{c}^{*}\left(\mathrm{nTR}\right)\sin\theta \end{array}\right]$$

Equations (5, 6) were used to analyze the relative abundance of mass‐labeled pyruvate and lactate in cells as observed by IC‐MS. Equations (7, 8) were used to analyze hyperpolarized, spin‐labeled pyruvate and lactate in cell suspensions by NMR.

PK model parameters were constrained to be non‐negative and fit to measured data using the trust region reflective nonlinear least squares optimization function lsqnonlin in Matlab (MathWorks, Natick, Massachusetts). To minimize the likelihood of settling into local minima, each dataset was fit at least 5000 times with parameter sets initialized at each repetition using random samples from a uniform distribution spanning beyond physiologically reasonable parameter values. The parameter set with the lowest mean square residual difference between measured and modeled data was selected as the best solution. In addition, to assess the uniqueness of parameter set solutions, each dataset was fit with *k*_*PL*_ values fixed and rastered over a range from 0.001 to 1.5 s^−1^ while all other unknown model parameters were allowed to vary. This approach identified a range of *k*_*PL*_ values that provided an essentially equivalent fit. We considered all parameter sets that yielded a residual that was reduced to within 1% of the minimum observed residual to be equivalent solutions, and we then selected the normalized parameter set with the lowest L2 norm to avoid parameter sets containing unrealistically large values for one or more parameters.

### 2.5 Patient Studies

A 66‐year old male with biopsy‐proven anaplastic thyroid cancer (ATC) of the left lobe of the thyroid was recruited to a prospective imaging study following IRB review and approval, FDA approval to administer HP pyruvate as an Investigational New Drug, and informed consent. This patient was scanned before start of treatment and again 8 days after starting systemic therapy (Pembrolizumab, Lenvatinib). Hyperpolarized pyruvate was prepared using a 5 T SpinLab dynamic nuclear polarization system (GE Healthcare, Waukesha WI). The patient was positioned head first and supine on a GE 3 T MR750 MRI scanner, with the thyroid positioned at approximately the center of a ^13^C “clamshell” transmit coil. A 4‐element “paddle” ^13^C receive array was positioned over the thyroid. Anatomic images were acquired using the body coil to confirm positioning and localize the tumor. Dynamic HP MRI data was acquired using a broadband EPI sequence with spectral‐spatial excitations [38], eight 8 mm‐thick slices, and a 16 × 16 image matrix covering a field of view of 24 cm × 24 cm. The acquisition was started just prior to injection of 250 mM HP pyruvate (0.43 mL/kg), and images of HP Pyruvate and lactate were acquired every 3 s for a total of 180 s. Dynamic data was reconstructed offline and analyzed using Matlab (Mathworks, Natick MA).

### 2.6 Pharmacokinetic Analysis of Dynamic HP MRI Data

The PK model described above for analysis of cells in vitro is easily extended (Figure 2B) for analysis of dynamic HP MRI data acquired in vivo. Equations (1, 2) are modified to account for HP pyruvate and lactate in the extravascular/extracellular space (EES):

(9) $$\begin{array}{ll} \frac{\delta {\mathrm{Pyr}}_{\mathrm{ee}}^{*}\left(t\right)}{\delta t} & =\frac{k_{\mathrm{ve}}}{v_{\mathrm{ee}}}\left({\mathrm{Pyr}}_{v}^{*}\left(t\right)-{\mathrm{Pyr}}_{\mathrm{ee}}^{*}\left(t\right)\right)-\frac{k_{\mathrm{ecP}}}{v_{\mathrm{ee}}}\left({\mathrm{Pyr}}_{\mathrm{ee}}^{*}\left(t\right)-{\mathrm{Pyr}}_{c}^{*}\left(t\right)\right)\\ & \;-R_{1,\mathrm{Pyr}}{\mathrm{Pyr}}_{m}^{*}\left(t\right) \end{array}$$

(10) $$\begin{array}{ll} \frac{\delta {\mathrm{Lac}}_{\mathrm{ee}}^{*}\left(t\right)}{\delta t} & =\frac{k_{\mathrm{ve}}}{v_{\mathrm{ee}}}\left({\mathrm{Lac}}_{v}^{*}\left(t\right)-{\mathrm{Lac}}_{\mathrm{ee}}^{*}\left(t\right)\right)-\frac{k_{\mathrm{ecL}}}{v_{\mathrm{ee}}}\left({\mathrm{Lac}}_{\mathrm{ee}}^{*}\left(t\right)-{\mathrm{Lac}}_{c}^{*}\left(t\right)\right)\\ & \;-R_{1,\mathrm{Lac}}{\mathrm{Lac}}_{m}^{*}\left(t\right) \end{array}$$

Here, ${\mathrm{Pyr}}_{v}^{*}\left(t\right)$ and ${\mathrm{Lac}}_{v}^{*}\left(t\right)$ represent vascular input functions for HP pyruvate and lactate and $k_{\mathrm{ve}}$ is the rate constant for flux of HP pyruvate between vascular space and EES. ${\mathrm{Pyr}}_{\mathrm{ee}}^{*}\left(t\right)$ and ${\mathrm{Lac}}_{\mathrm{ee}}^{*}\left(t\right)$ are substituted for ${\mathrm{Pyr}}_{m}^{*}\left(t\right)$ and ${\mathrm{Lac}}_{m}^{*}\left(t\right)$ in Equations (3, 4) and the solution to the system of equations becomes

(11) $$\begin{array}{cc} \left[\begin{array}{c} {\mathrm{Pyr}}_{\mathrm{ee}}^{*}\left(\mathrm{nTR}\right)\\ {\mathrm{Lac}}_{\mathrm{ee}}^{*}\left(\mathrm{nTR}\right)\\ {\mathrm{Pyr}}_{c}^{*}\left(\mathrm{nTR}\right)\\ {\mathrm{Lac}}_{c}^{*}\left(\mathrm{nTR}\right) \end{array}\right] & =e^{A_{3}\cdot \mathrm{TR}}\left[\begin{array}{c} {\mathrm{Pyr}}_{\mathrm{ee}}^{*}\left(\left[n-1\right]\mathrm{TR}\right)\cos\theta_{P}\\ {\mathrm{Lac}}_{\mathrm{ee}}^{*}\left(\left[n-1\right]\mathrm{TR}\right)\cos\theta_{L}\\ {\mathrm{Pyr}}_{c}^{*}\left(\left[n-1\right]\mathrm{TR}\right)\cos\theta_{P}\\ {\mathrm{Lac}}_{c}^{*}\left(\left[n-1\right]\mathrm{TR}\right)\cos\theta_{L} \end{array}\right]\\ & \;+\frac{k_{\mathrm{ve}}}{v_{\mathrm{ee}}}\int_{\left[n-1\right]\mathrm{TR}}^{\mathrm{nTR}}e^{A_{3}\cdot \left(\mathrm{nTR}-\tau \right)}\left[\begin{array}{c} {\mathrm{Pyr}}_{v}^{*}\left(\tau \right)\\ {\mathrm{Lac}}_{v}^{*}\left(\tau \right)\\ 0\\ 0 \end{array}\right]d\tau \end{array}$$

where

(12) $$\begin{array}{cc} & A_{3}=\left[\begin{array}{cccc} -\;\left(\frac{k_{\mathrm{ve}}}{v_{\mathrm{ee}}}+\frac{k_{\mathrm{ecP}}}{v_{\mathrm{ee}}}+R_{1,\mathrm{Pyr}}\right) & 0 & \frac{k_{\mathrm{ecP}}}{v_{\mathrm{ee}}} & 0\\ 0 & -\left(\frac{k_{\mathrm{ve}}}{v_{\mathrm{ee}}}+\frac{k_{\mathrm{ecL}}}{v_{\mathrm{ee}}}+R_{1,\mathrm{Lac}}\right) & 0 & \frac{k_{\mathrm{ecL}}}{v_{\mathrm{ee}}}\\ \frac{k_{\mathrm{ecP}}}{v_{c}} & 0 & -\left(\frac{k_{\mathrm{ecP}}}{v_{c}}+k_{\mathrm{PL}}+R_{1,\mathrm{Pyr}}\right) & k_{\mathrm{LP}}\\ 0 & \frac{k_{\mathrm{ecL}}}{v_{c}} & k_{\mathrm{PL}} & -\left(\frac{k_{\mathrm{ecL}}}{v_{c}}+k_{\mathrm{LP}}+R_{1,\mathrm{Lac}}\right) \end{array}\right]. \end{array}$$

The total observed signal is then given by

(13) $$\begin{array}{cc} \left[\begin{array}{c} {\mathrm{Pyr}}_{T}^{*}\left(\mathrm{nTR}\right)\\ {\mathrm{Lac}}_{T}^{*}\left(\mathrm{nTR}\right) \end{array}\right] & =v_{b}\left[\begin{array}{c} {\mathrm{Pyr}}_{v}^{*}\left(\mathrm{nTR}\right)\sin\theta_{P}\\ {\mathrm{Lac}}_{v}^{*}\left(\mathrm{nTR}\right)\sin\theta_{L} \end{array}\right]+v_{\mathrm{ee}}\left[\begin{array}{c} {\mathrm{Pyr}}_{m}^{*}\left(\mathrm{nTR}\right)\sin\theta_{P}\\ {\mathrm{Lac}}_{m}^{*}\left(\mathrm{nTR}\right)\sin\theta_{L} \end{array}\right]\\ & \;+v_{c}\left[\begin{array}{c} {\mathrm{Pyr}}_{c}^{*}\left(\mathrm{nTR}\right)\sin\theta_{P}\\ {\mathrm{Lac}}_{c}^{*}\left(\mathrm{nTR}\right)\sin\theta_{L} \end{array}\right] \end{array}$$

In this study, the vascular input function was identified in a nearby artery and no HP lactate signal was observed. Each voxel that met criteria for analysis was analyzed using the same approach described above, with controlled rasterization of $k_{\mathrm{PL}}$ values, repeated fits with random initial seeds, and ultimate selection of the parameter set with the lowest L2 norm among practically equivalent solutions. Upper bounds for $k_{\mathrm{LP}}$, $k_{\mathrm{ecP}}$, and $k_{\mathrm{ecL}}$ in vivo were set to extend beyond values observed by HP NMR in vitro, excluding obvious outliers, by a factor of five or more to allow for effects of differences between the two environments.

This PK model is composed of three physical compartments (3PC) and only allows chemical conversion of HP pyruvate into lactate within intracellular space, at an apparent rate constant identified as $k_{\mathrm{PL}}$. The model can be reduced in complexity by assuming that extravascular space is well‐mixed and considering only two physical compartments (2PC) [16, 37], with the apparent conversion rate denoted as $k_{\mathrm{PL}}^{\prime }$ because it is derived from a reduced model and affected by additional factors such as the cell volume fraction and rate of transport across the cell membrane, and thus does not uniquely describe intracellular pyruvate metabolism [12]. Further simplification to a precursor‐product model with a single physical compartment yields $k_{\mathrm{PL}}^{\prime \prime }$ which is also affected by vascular function. Repeated fits and rastering of $k_{\mathrm{PL}}^{\prime \prime }$ is not necessary when using the precursor‐product model. Table 1 summarizes the parameters that were used in these analyses.

**TABLE 1:** Parameters and parameter constraints that were used in the analysis of dynamic HP MRI data. T1 values for pyruvate and lactate were assumed to be constant at 64 and 55 s, respectively, based on prior studies indicating that consistent over‐estimation of these values does not significantly affect accuracy or reproducibility. For 2PC and 3PC analyses, *k*_*PL*_ values were assumed as known values and rastered over the range defined above, while remaining parameters were fit to the data. To minimize likelihood of falling in local minima, fits were repeated 5000 times per *k*_*PL*_ value using random seed points within the defined ranges. The shape of the VIF was derived from an artery near the tumor, while the scale factor of the VIF was fit. For 2PC, *v*_*e*_ = 1−*v*_*b*_ and for 3PC, *v*_*ee*_ = *v*_*ef*_ × (1−*v*_*b*_) and *v*_*c*_ = 1−*v*_*b*_−*v*_*ee*_.

Model	Parameter name	Unit	Lower bound	Upper bound
Precursor‐product (1PC)	$k_{\mathrm{PL}}^{\prime \prime }$	s^−1^	0	∞
Two‐compartment (2PC)	$k_{\mathrm{PL}}^{\prime }$	s^−1^	0.01	1.0
	$k_{\mathrm{LP}}^{\prime }$	s^−1^	10^−5^	1.0
	VIF scale		0	∞
	$k_{\mathrm{ve}}$	s^−1^	0.001	0.2
	$v_{b}$		0.001	1.0
Three‐compartment (3PC)	$k_{\mathrm{PL}}$	s^−1^	0.1	2.0
	$k_{\mathrm{LP}}$	s^−1^	10^−5^	2.0
	VIF scale		0	∞
	$k_{\mathrm{ve}}$	s^−1^	0.001	0.2
	$v_{b}$		0.001	1.0
	$k_{\mathrm{ecP}}$	s^−1^	0.001	0.5
	$k_{\mathrm{ecL}}$	s^−1^	10^−5^	1.0
	$v_{\mathrm{ef}}$		0.2	0.8

### 2.7 Statistical Analyses

All data were analyzed using Matlab. Three technical replicates of IC‐MS from each cell line at each exposure time were averaged to generate pseudo‐dynamic mass spec data. Linear regression between *k*_*PL*_, the apparent rate constant for conversion of labeled pyruvate into lactate, derived from dynamic HP NMR data versus derived from pseudo‐dynamic IC‐MS data across three cell lines, was carried out using the fitlm function. The single value of *k*_*PL*_ derived from IC‐MS data acquired from each cell line was treated as the independent variable, while multiple replicates of Hth83 (*N* = 5), PC3 (*N* = 3), and LnCap (*N* = 4) were treated as dependent variables. The F‐statistic was calculated to compare the linear model against the null hypothesis that *k*_*PL*_ derived from HP NMR could be adequately represented by the mean value of all measurements (*N* = 12).

Metrics of HP pyruvate metabolism from patient scans were compared using a one‐sided Wilcoxon rank sum test, and the effect size was calculated using Cohen's d with a pooled standard deviation. The normalized lactate ratio (nLac) and apparent rate constants for conversion of HP pyruvate to lactate were derived from HP MRI voxels that contained at least 40% tumor in cross‐section based on anatomical reference images at the center of each HP MRI slice. Non‐enhancing voxels within the tumor ROI were discarded. At least 30 enhancing voxels from each timepoint were used to test whether the distributions of nLac, $k_{\mathrm{PL}}^{\prime \prime }$, $k_{\mathrm{PL}}^{\prime }$, and $k_{\mathrm{PL}}$ were different after 8 days of treatment compared to baseline.

## 3 Results

### 3.1 Quantifying *k*_*PL*_ by Dynamic Incorporation of M + 3‐Labeled Pyruvate With IC‐MS

**FIGURE 3:**
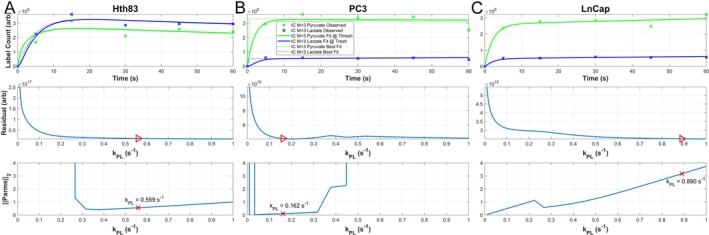
Pharmacokinetic analysis of [U‐^13^C_3_]‐pyruvate metabolism using pseudo‐dynamic IC‐MS. Top row demonstrates agreement between measured and modeled data from (A) Hth‐83 anaplastic thyroid, (B) PC3 prostate, and (C) LnCap prostate cancer cells exposed to ˜1.0 mmol/L HP [1‐^13^C]‐pyruvate. The PK model with two physical compartments (intracellular, extracellular space) and two chemical pools (pyruvate and lactate) fit the data with *k*_*PL*_ = 0.559, 0.162, and 0.890 s^−1^, for Hth83, PC3, and LnCap cells, respectively. Middle row: To assess the uniqueness of the solution, nuisance parameters were estimated while *k*_*PL*_ was varied over a supraphysiological range. A range of equivalent solutions is identified beginning with the lowest value for *k*_*PL*_ at which the residual curve is reduced to within 1% of its minimum (red triangle) from the maximum observed residual difference. Bottom row: Within the range of equivalent solutions, the *k*_*PL*_ value associated with the lowest L2‐norm of model parameters is selected (red “x”).

Hth‐83, PC‐3, and LnCap cells were exposed to normal media enriched with 1 mM [U‐^13^C_3_]‐pyruvate for up to 60 s (see Figure 1), with metabolite labeling measured via IC‐MS. Mass labels in the intracellular pyruvate and lactate pools were observed at ˜70% of their equilibrium levels after only 5 s exposure to labeled pyruvate in media (Figure 3), a finding that is also supported by NMR spectroscopy (see Figure S1). By controlling *k*_*PL*_, the apparent rate constant for conversion of HP pyruvate to lactate, and fitting the remaining model parameters at each *k*_*PL*_ value, a range of virtually equivalent parameter sets were identified that closely match the measured data, with a residual difference that is within 1% of the minimum observed residual. Selection of the parameter set with the lowest L2 norm among approximately equivalent parameter sets yields *k*_*PL*_ = 0.559, 0.162, and 0.890 s^−1^, for Hth‐83, PC3, and LnCap cancer cells, respectively.

### 3.2 PK Analysis of HP Pyruvate Metabolism Corresponds With IC‐MS Across Cell Lines

**FIGURE 4:**
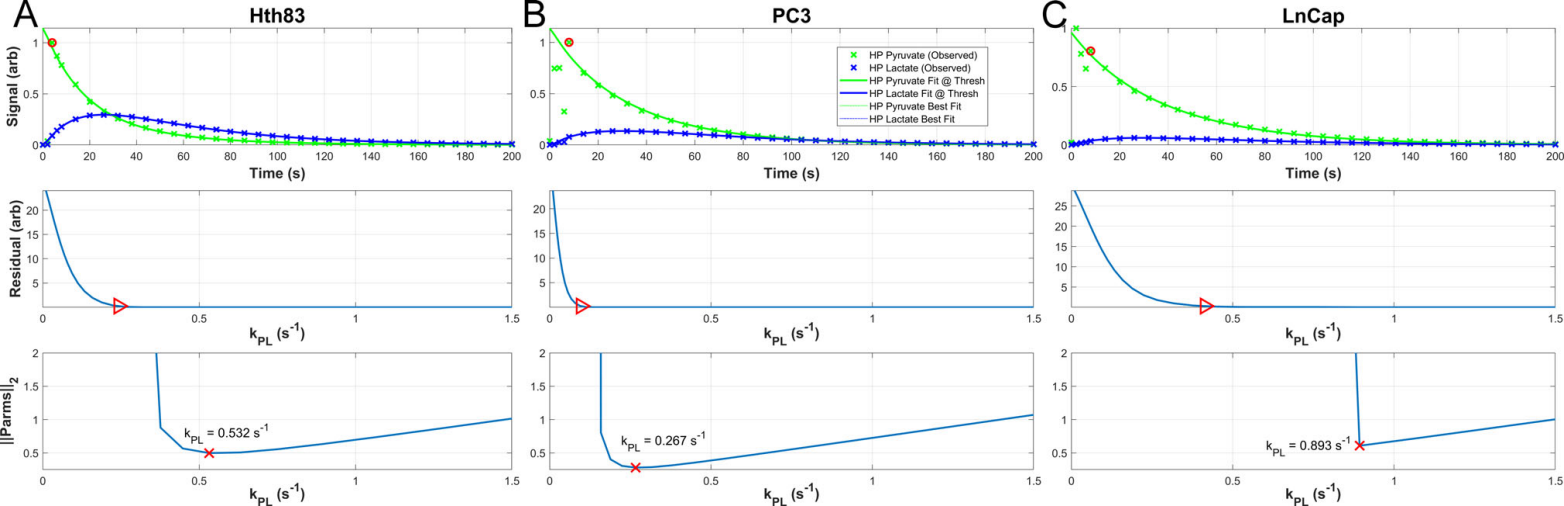
Pharmacokinetic analysis of HP [1‐^13^C]‐pyruvate metabolism using dynamic NMR. Top row demonstrates agreement between measured and modeled data from (A) Hth‐83 anaplastic thyroid, (B) PC3 prostate, and (C) LnCap prostate cancer cells exposed to ˜3.07 mmol/L HP [1‐^13^C]‐pyruvate. The PK model with two physical compartments (intracellular, extracellular space) and two chemical pools (pyruvate and lactate) fit the data from these representative samples with *k*_*PL*_ = 0.532, 0.267, and 0.893 s^−1^, for Hth83, PC3, and LnCap cells, respectively. The red circle represents the point at which signal stabilized after the addition of HP pyruvate, and the starting point of the data that was fit to the model. Middle row: To assess the uniqueness of the solution, nuisance parameters were estimated while *k*_*PL*_ was varied over a supraphysiological range. A range of equivalent solutions is identified beginning with the lowest value for *k*_*PL*_ at which the residual curve is reduced to within 1% of its minimum (red triangle) from the maximum observed residual difference. Bottom row: Within the range of equivalent solutions, the *k*_*PL*_ value associated with the lowest L2‐norm of model parameters is selected (red “x”).

**FIGURE 5:**
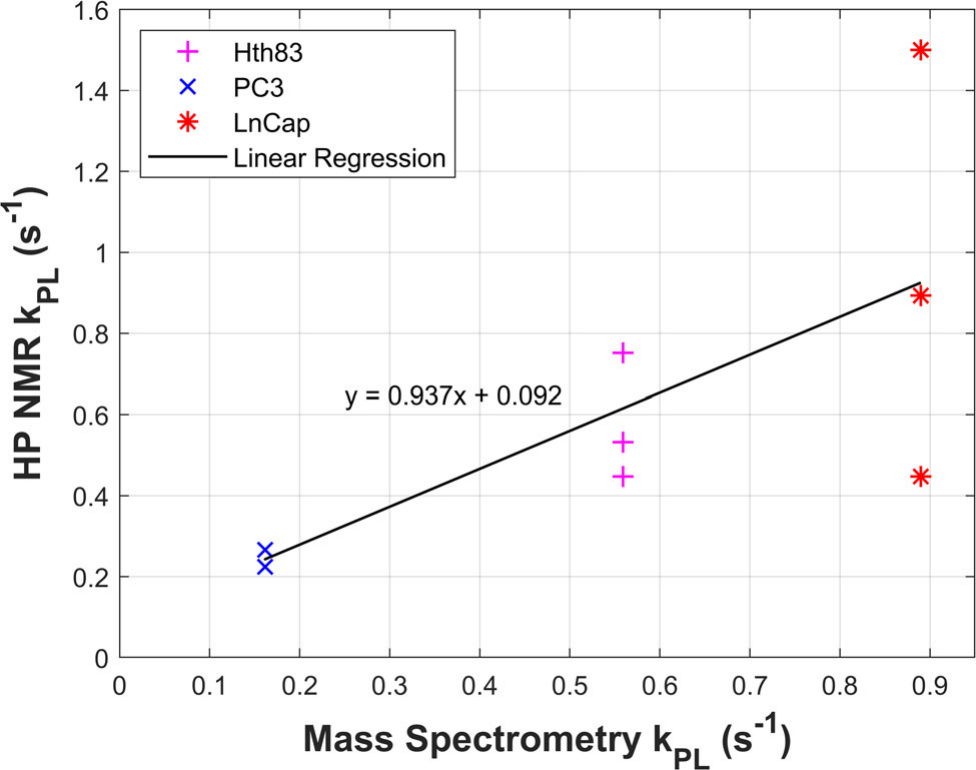
*k*_*PL*_ values derived from dynamic NMR of HP [1‐^13^C]‐pyruvate correspond with *k*_*PL*_ values derived from pseudo‐dynamic IC‐MS of [U‐^13^C_3_]‐pyruvate. Five samples from each cell line were obtained from IC‐MS after exposure to [U‐^13^C_3_]‐pyruvate for 0–60s to yield a single value, while three to five samples from each cell line yielded unique values of *k*_*PL*_ from NMR data. Grouped data fits to a first‐order linear model (*p* = 0.005).

Dynamic NMR spectroscopy following exposure to approximately 3 mM HP [1‐^13^C]‐pyruvate was also used to assess pyruvate metabolism in Hth‐83, PC‐3, and LnCap cells (Figure 4). PK analysis of this data also revealed a range of virtually equivalent solutions, and by selecting the parameter sets with the lowest L2 norm, we found that by NMR, with 3–5 replicates from each cell line, *k*_*PL*_ = 0.603 ± 0.14 s^−1^, 0.252 ± 0.024 s^−1^, and 0.934 ± 0.432 s^−1^ for Hth‐83, PC3, and LnCap cancer cells, respectively. Regression of *k*_*PL*_ values obtained from NMR on IC‐MS values (Figure 5) supports a linear relationship (*p* = 0.005) with a slope of 0.937 and a 90% confidence interval of (0.345, 1.528). Both approaches identify LnCap cells as having the fastest rate of pyruvate metabolism under these experimental conditions, followed by Hth‐83 and finally PC3 with the slowest rate of pyruvate metabolism.

### 3.3 Assessment of Tumor Metabolism and Metabolic Heterogeneity With HP‐Pyruvate MRI

**FIGURE 6:**
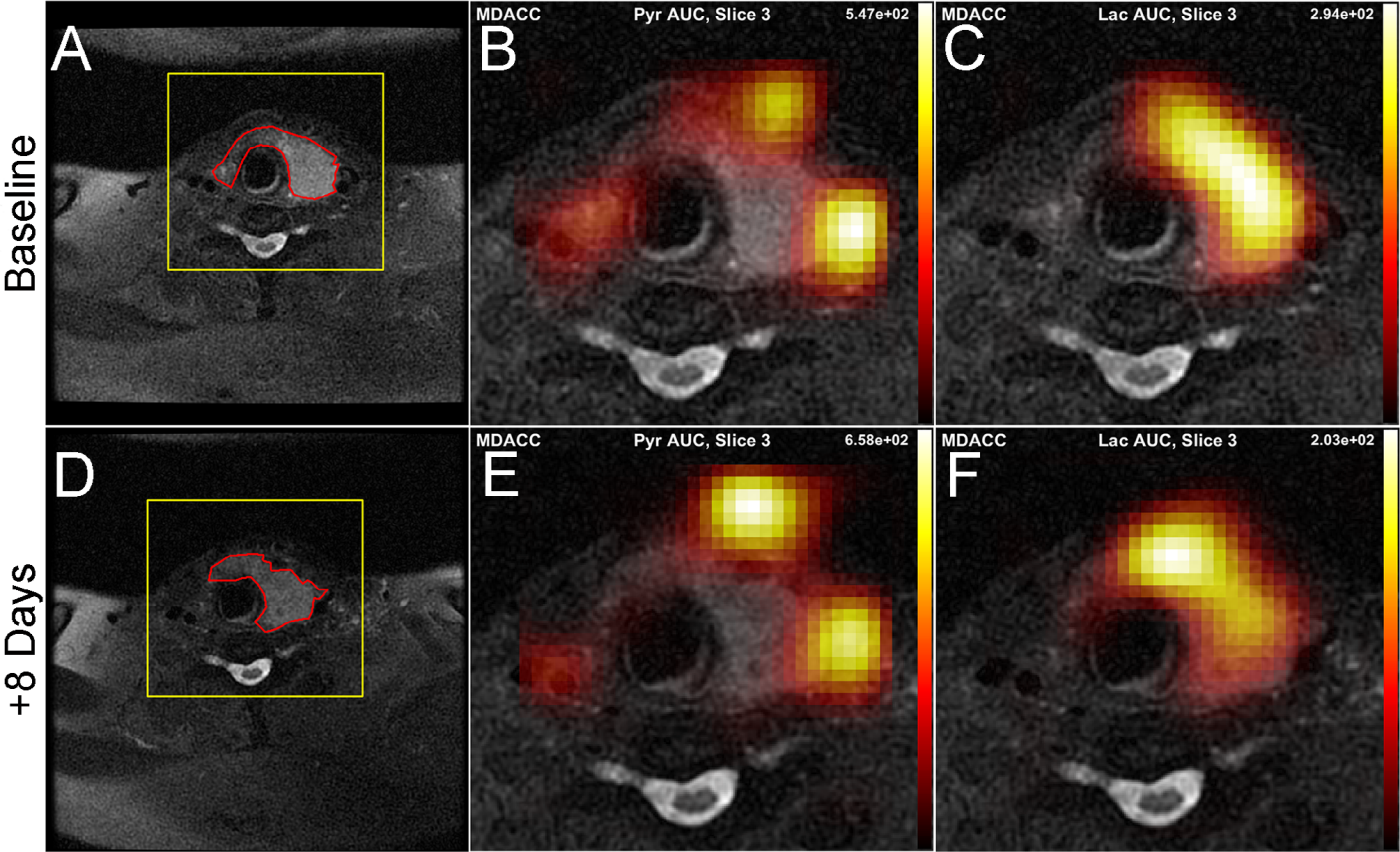
Dynamic spectroscopic imaging of hyperpolarized [1‐13C]‐pyruvate provides a minimally invasive mechanism for visualizing pyruvate metabolism in vivo. This patient with biopsy‐proven anaplastic thyroid cancer (ATC) was scanned prior to start of induction chemotherapy (A–C), and again after 8 days of treatment with Lenvatinib and Pembrolizumab (D–F). The left column (A, D) shows the anatomic reference image with the tumor outlined in red. The center column (B, E) shows a strong signal from pyruvate at both timepoints, and the rightmost column (C, F) reveals hyperpolarized lactate that can only be formed by the interaction of HP pyruvate with intracellular enzymes. Images are individually scaled to maximum intensity for visualization, with the scale factor shown in the upper right of each panel. The signal from HP lactate is markedly lower after 8 days of treatment.

Hyperpolarized [1‐^13^C]‐pyruvate was administered by intravenous injection to a patient with anaplastic thyroid cancer prior to and after 8 days of treatment with Pembrolizumab and Lenvatinib. HP pyruvate was detectable in tumor tissue and in nearby vessels. HP lactate, which can only arise through interaction of injected HP pyruvate with intracellular LDH and NADH, was observed in tumor tissue (Figure 6). The normalized lactate ratio (nLac = AUC_L_ /[AUC_L_ + AUC_P_]) observed in tumor voxels was lower following 8 days of treatment (mean baseline versus follow‐up: 0.361 vs. 0.311, Cohen's d = 0.29; median: 0.418 vs. 0.315; max: 0.684 vs. 0.660), suggesting that treatment induced an overall reduction in tumor metabolism, though the difference is not statistically significant (*p* = 0.125). Analysis using a simple precursor‐product PK model suggests a larger and statistically significant metabolic change ($k_{\mathrm{PL}}^{\prime \prime }$ mean: 0.033 s^−1^ vs. 0.018 s^−1^, Cohen's d = 0.67; median: 0.034 s^−1^ vs. 0.017 s^−1^; max: 0.115 s^−1^ vs. 0.045 s^−1^; *p* = 0.032).

### 3.4 An Extended PK Model Estimates Intracellular Metabolism in Human Subjects

AUC metrics (and AUC ratios) offer no ability to distinguish signals arising from vasculature versus those from extravascular/extracellular or intracellular space, and are less specific to intracellular metabolism. Therefore, the reduction at 8 days observed in the normalized lactate AUC ratio and simplified precursor product pharmacokinetic analysis ($k_{\mathrm{PL}}^{\prime \prime }$) cannot be specifically attributed to metabolic changes alone. Indeed, vascular changes were observed using dynamic, contrast‐enhanced (DCE‐) MRI measurements [39]. Significant reductions in DCE‐MRI parameters including *v*_*b*_, the vascular blood volume fraction, and *K* ^*trans*^, the rate constant for extravasation of the MRI contrast agent (Figure S2) were observed at 8 days compared to baseline.

PK models of pyruvate metabolism that account for signals from vasculature separately from signals that arise from extravascular/extracellular or intravascular compartments seek to reduce bias imparted by vascular function on metabolic biomarkers. PK analysis using a two‐compartment (2PC) model that separates vascular signal from a well‐mixed extravascular space [16] suggests a more complex and heterogeneous response with reduced pyruvate extravasation ($k_{\mathrm{ve}}$, *p* = 0.009) and metabolic changes that are not statistically significant ($k_{\mathrm{PL}}^{\prime }$ mean: 0.140 s^−1^ vs. 0.123 s^−1^, Cohen's d = 0.14; median: 0.127 s^−1^ vs. 0.091 s^−1^; max: 0.641 s^−1^ vs. 0.655 s^−1^; *p* = 0.17). With vascular effects accounted for by separate model parameters, this analysis suggests that pyruvate metabolism in enhancing voxels is modestly lower on average, but that the maximum rate of pyruvate metabolism across the tumor has increased.

**FIGURE 7:**
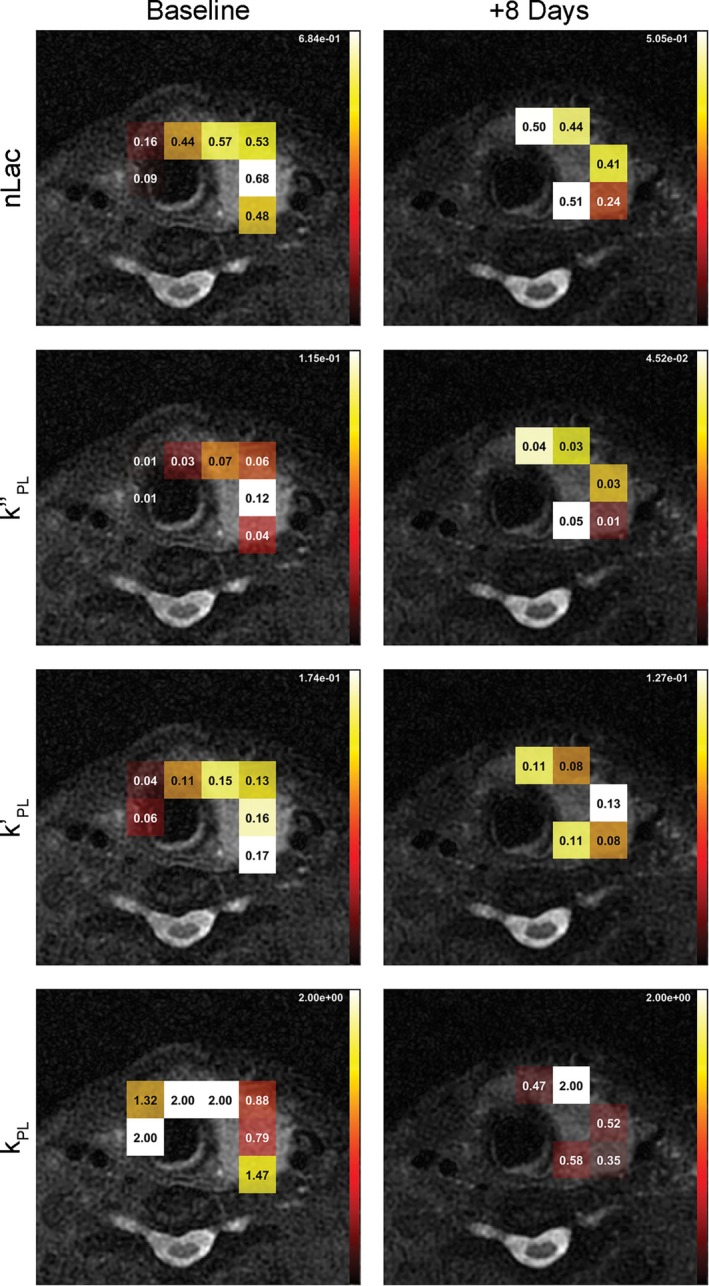
Semi‐quantitative and quantitative measures of HP pyruvate metabolism in a patient with anaplastic thyroid cancer show contrasting metabolic response. The normalized lactate ratio (nLac) shows that pyruvate metabolism in enhancing voxels is globally lower after 8 days of treatment, but the difference is not statistically significant. The precursor‐product model with one physical compartment shows a statistically significant reduction in $k_{\mathrm{PL}}^{\prime \prime }$ (*p* = 0.032). A two‐compartment model that separates vascular from extravascular signals, and thus corrects for changes in vasculature, shows a significant reduction in HP pyruvate extravasation ($k_{\mathrm{ve}}$, *p* = 0.009) and a heterogeneous response with slightly lower average $k_{\mathrm{PL}}^{\prime }$ after 8 days. The three‐compartment model and analytical approach that was built from in vitro cell culture studies identifies regions of the tumor with extremely rapid pyruvate metabolism (capped at 2.0 s^−1^) and significant reduction in average $k_{\mathrm{ve}}$ (*p* = 0.02) and $k_{\mathrm{PL}}$ (*p* = 0.017) across the tumor ROI. The histograms for metrics of pyruvate metabolism are shown in Figure S3.

Each voxel within the tumor ROI was analyzed using the proposed 3PC model and the approach summarized in Figure 4. This model paints a similarly complex picture of metabolic response to therapy with lower $k_{\mathrm{ve}}$ (*p* = 0.02) and metabolic activity significantly lower on average (*p* = 0.017) in enhancing voxels across the tumor ($k_{\mathrm{PL}}$ mean: 1.30 s^−1^ vs. 0.932 s^−1^, Cohen's d = 0.53; median: 1.32 s^−1^ vs. 0.579 s^−1^; max: 2.0 s^−1^ vs. 2.0 s^−1^). At both pre‐ and post‐treatment timepoints, however, some regions of the tissue show conversion of HP pyruvate to lactate fitting to the highest rate considered during analysis. Figure 7 summarizes a representative slice of data acquired at baseline and + 8 days after therapy using each of these approaches to analysis of dynamic HP MRI data, with histograms shown in Figure S3.

## 4 Discussion

Hyperpolarized pyruvate provides unique insight into the biochemistry of tumor metabolism in vivo, and shows tremendous potential for giving new insight into early metabolic changes that are induced by cancer therapy. Quantification of the spatial and chemical fate of HP pyruvate is challenging because of its relatively short observable lifetime and complex interaction with multiple physical barriers prior to interaction with enzymes that mediate conversion of HP pyruvate into lactate. The most widely used metrics of HP pyruvate metabolism, such as the AUC_L_:AUC_P_ ratio, the normalized lactate ratio, and simplified precursor‐product PK models, are straightforward to calculate but are biased by a variety of factors that are peripheral to intracellular metabolism and interaction with metabolic enzymes that can uniquely be revealed by HP pyruvate.

In this work, we developed a PK model for quantification of HP pyruvate metabolism that shows good correspondence with quantification of mass‐labeled pyruvate metabolism as observed using IC‐MS. Metabolic tracing of stable isotopes by MS has long been used to study the biochemistry of metabolism in vitro and in vivo, and is regarded here as the gold standard against which quantification of spin‐labeled pyruvate metabolism can be compared. By measuring *k*_*PL*_, the apparent rate constant for conversion of labeled pyruvate into lactate, in three cell lines by HP MRS and by IC‐MS, we confirm a statistically significant linear relationship between the *k*_*PL*_ values that are derived from HP MRS and those that are derived from pseudo‐dynamic IC‐MS. The use of a higher nominal concentration of labeled pyruvate for HP NMR compared to IC‐MS may have led to slightly lower estimates of *k*_*PL*_ observed by HP NMR relative to IC‐MS, however, the slope of the linear relationship between *k*_*PL*_ values derived from the two approaches (Figure 5) is near unity and has a relatively large confidence interval. Matched concentrations may have yielded a different slope but would not be expected to alter the rank order of metabolic activity for either approach. The framework for analyzing this data by sweeping $k_{\mathrm{PL}}$ values across a super‐physiological range and repeated fitting of the remaining model parameters recognizes a range of virtually equivalent parameter sets, and the use of the L2 norm of normalized model parameters to select a $k_{\mathrm{PL}}$ value within that range avoids parameter sets that include unrealistically large values of parameters that compensate for misestimation of other model parameters. This approach also reduces the potential for $k_{\mathrm{PL}}$ to be selected at a local minimum that could be strongly influenced by the seed point of the optimization.

The validated PK model and framework for analysis of HP pyruvate metabolism in vitro is easily extended to form a rationally designed model with three physical compartments (intravascular, extravascular/extracellular, and intracellular) that can be used for quantitative analysis of HP pyruvate metabolism in vivo. This model was used to evaluate tumor metabolism in a patient with anaplastic thyroid cancer, a rare but extremely aggressive subtype of thyroid cancer, at baseline prior to the start of induction chemotherapy and again after 8 days of treatment with Pembrolizumab and Lenvatinib. Lenvatinib is a multi‐VEGFR kinase inhibitor that induced a statistically significant change in tumor microvascular function, as confirmed by DCE‐MRI. While the precursor‐product metric of tumor metabolism ($k_{\mathrm{PL}}^{\prime \prime }$) suggested a statistically significant reduction in tumor metabolism across the tumor, it is impossible to know whether this difference was primarily influenced by changes in intracellular metabolism, transport, cell density, or vasculature. The 2PC and 3PC models separately account for vascular function. The 2PC model assumes a well‐mixed extravascular compartment, and $k_{\mathrm{PL}}^{\prime }$ derived from 2PC PK analysis reflects a combination of cell density, transport, and intracellular metabolism. The 3PC model separately accounts for cell density and transport, and $k_{\mathrm{PL}}$ derived from 3PC PK analysis uniquely reflects intracellular pyruvate metabolism. Analysis using the proposed approach confirmed an overall reduction of intracellular pyruvate metabolism across the tumor ROI, and it also identified regions of the tumor with extremely high metabolic activity; the maximum value of *k*_*PL*_ observed over the whole tumor ROI was not reduced after 8 days of treatment. In several voxels with extremely high metabolic activity, the “plateau” of nonunique solutions was not reached within the range of *k*_*PL*_ values considered, and it is plausible that in those regions, metabolic activity may be too fast to be accurately quantified by this approach. The ability to identify and specifically target unresponsive regions such as this could present new opportunities for personalized treatment of aggressive cancers.

HP pyruvate is currently being evaluated in clinical studies at more than a dozen leading institutions around the world. Clinical adoption of HP MRI to guide care for patients will require conclusive demonstration in larger trials in order to show that HP metabolic imaging biomarkers are robust, reproducible, and specific, with a clear association to clinical outcomes. The three‐compartment model used in this work seeks to reduce bias that can be imparted by changes in vascular function, transport across the cell membrane, and cellular density. A central limitation of this work is the modest number of cell lines that were analyzed in vitro, the application of the 3PC model to data from a single subject, and the absence of pathological correlates. Further studies are planned to evaluate more cell lines in vitro and under a range of therapeutic conditions, to characterize and reduce uncertainty in quantification, and to leverage this model in ongoing patient studies. The model and framework for analysis may evolve as new information arises from additional studies in vitro, in animal models, and in patients with cancer. Full clinical adoption of HP MRI is also limited by the number of sites with MRI scanners that are configured for multinuclear acquisitions and the dearth of coils that are optimized for ^13^C while supporting standard‐of‐care quality anatomic and functional MRI. Advances in polarizer technology could also significantly reduce the barrier to routine use of HP MRI in the clinical setting. Recent FDA approval of HP ^129^Xe MRI for imaging lung ventilation illustrates the promise for clinical adoption of metabolic MRI using HP pyruvate.

## Funding

This work was supported by the National Cancer Institute (P30CA016672, R01CA211150, R01CA280980, U54CA274321), GE Healthcare, and the Cancer Prevention and Research Institute of Texas (RP170366, RP210028).

## Conflicts of Interest

Dr. Bankson serves on the scientific advisory board for NVision Imaging Technologies GmbH, and he receives research funding from Siemens Medical Solutions USA Inc. Dr. Lai serves as a medical affairs consultant for Cardinal Health. Dr. Sandulache is a consultant for FemtoVox Inc.

## Supporting information


**Figure S1:** 1H NMR to estimate the fractional enrichment at the C3 of lactate after 5 s exposure to [U‐^13^C_3_]‐pyruvate.
**Figure S2:** *K* ^*trans*^ maps from dynamic contrast enhanced imaging acquired after the hyperpolarized images of a patient with anaplastic thyroid cancer. The *K* ^*trans*^ maps prior to therapy show a well perfused thyroid tumor (left). *K* ^*trans*^ maps 8 days after initiation of therapy show a poorly perfused tumor (right) indicating a significant vascular response to therapy.
**Figure S3:** Histograms of HP MRI metrics in the tumor ROI at baseline and after + 8 days on therapy. The normalized lactate ratio (nLac) reflects the area‐under‐the‐curve (AUC) for lactate divided by the sum of AUCs for pyruvate and lactate. $k_{\mathrm{PL}}^{\prime \prime }$ is the apparent rate constant for conversion of HP pyruvate into lactate when quantified using a precursor‐product phamarokinetic (PK) model. For a PK model with two physical compartments (vascular, extravascular), $k_{\mathrm{PL}}^{\prime }$ reflects the apparent rate constant for conversion of HP pyruvate into lactate in a well‐mixed extravascular environment. In the three‐compartment model that accounts for intravascular, extravascular/extracellular, and intracellular space separately, $k_{\mathrm{PL}}$ reflects the apparent rate constant for intracellular HP pyruvate metabolism.

## Data Availability

The data generated in this study are available from the corresponding author upon reasonable request. Matlab scripts that were used for data analysis and for creation of the figures that appear within this manuscript are publicly available through the following link: https://github.com/mda‐mrsl/QHPMRIvMS/tree/64f22d7.
