# Supplementary material for: Quantitative Imaging of Pyruvate Metabolism in a Patient With Anaplastic Thyroid Cancer
Source: Magn Reson Med. 2026 Mar 24;96(2):826–38. doi: 10.1002/mrm.70351 (PMC13269194; doi:10.1002/mrm.70351)
Supplement: Supplementary file 1 — Figure S1: 1H NMR to estimate the fractional enrichment at the C3 of lactate after 5 s exposure to [U‐13C3]‐pyruvate. Figure S2: K trans maps from dynamic contrast enhanced imaging acquired after the hyperpolarized images of a patient with anaplastic thyroid cancer. The K trans maps prior to therapy show a well perfused thyroid tumor (left). K trans maps 8 days after initiation of therapy show a poorly perfused tumor (right) indicating a significant vascular response to therapy. Figure S3: Histograms of HP MRI metrics in the tumor ROI at baseline and after + 8 days on therapy. The normalized lactate ratio (nLac) reflects the area‐under‐the‐curve (AUC) for lactate divided by the sum of AUCs for pyruvate and lactate. kPL′′ is the apparent rate constant for conversion of HP pyruvate into lactate when quantified using a precursor‐product phamarokinetic (PK) model. For a PK model with two physical compartments (vascular, extravascular), kPL′ reflects the apparent rate constant for conversion of HP pyruvate into lactate in a well‐mixed extravascular environment. In the three‐compartment model that accounts for intravascular, extravascular/extracellular, and intracellular space separately, kPL reflects the apparent rate constant for intracellular HP pyruvate metabolism. [file MRM-96-826-s001.docx]

**Supplementary Figure S1**

Hth83 anaplastic thyroid cells were prepared as described for IC-MS measurements above, and incorporation of the 13C label into lactate after 5s exposure to [U-^13^C_3_]-pyruvate was also assessed by NMR. ^1^H NMR spectra (Fig. S1) were acquired at 14.1 T using a Bruker 1.7 mm HTS , ^1^H-^13^C cryoprobe (Bruker, Billerica, MA, USA). To estimate the fractional enrichment at the C3 of lactate, interleaved 1H spectra were acquired with ^13^C broadband GARP decoupling either on or off. Difference spectra produce a quantitative readout of ^13^C enrichment by monitoring the methyl signal of lactate at 1.33 ppm. This method is extremely sensitive to enrichment and can estimate fractional changes in incorporation below 1%. In this case, approximately 37% of the intracellular lactate pool was labeled with ^13^C after 5s exposure to [U-^13^C_3_]-pyruvate .


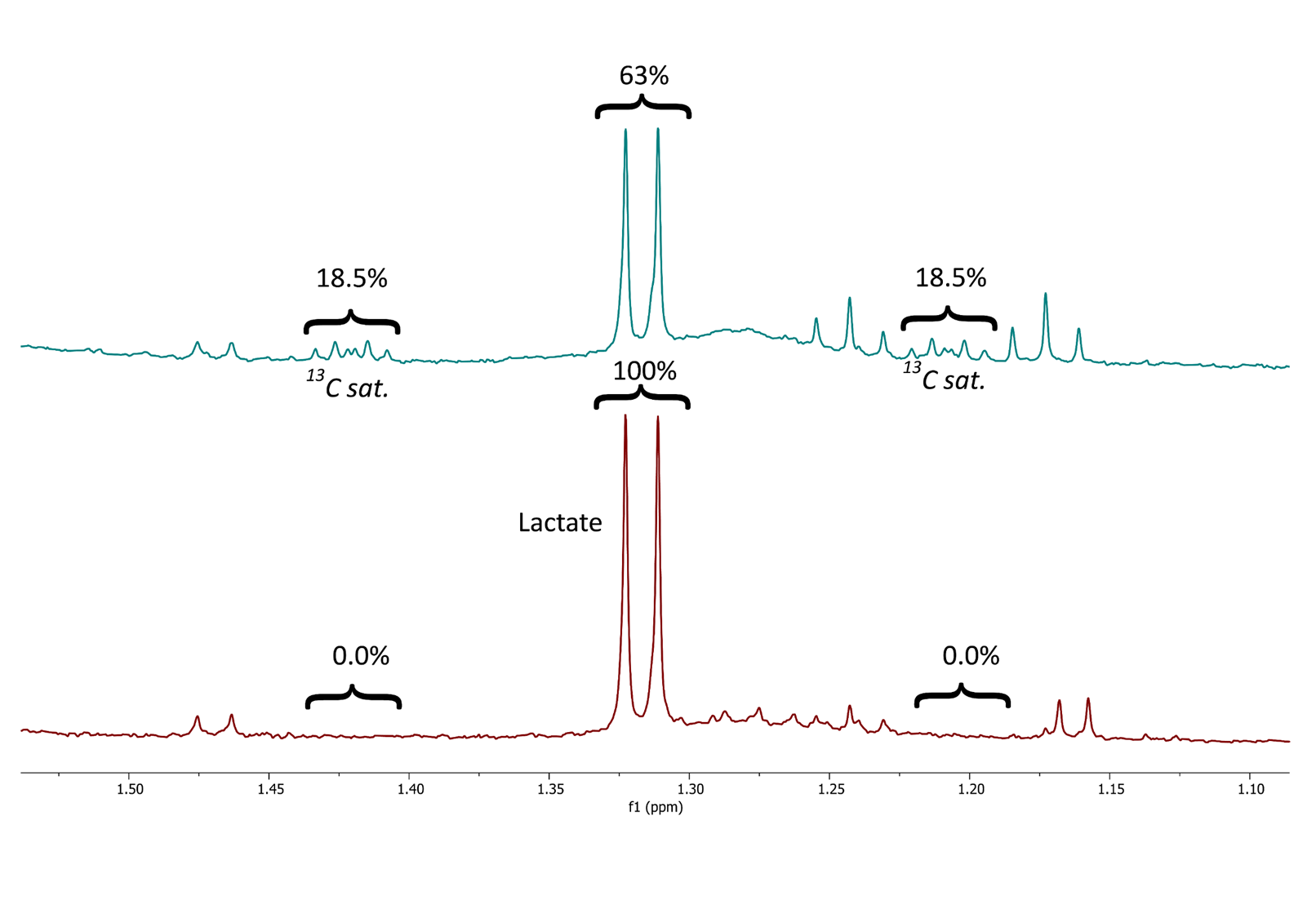


**Figure S1.** 1H NMR to estimate the fractional enrichment at the C3 of lactate after 5s exposure to [U-^13^C_3_]-pyruvate.

**Supplementary Figure S2**

After HP MRI, the patient was removed from the table and coils were changed to those used for standard of care imaging of the head and neck. Advanced proton MRI acquisitions were performed, including T_1_-weighted, T_2_-weighted, and diffusion weighted images as well as dynamic contrast enhanced (DCE) MRI images. DCE data was acquired using a 3D gradient echo with 2x2x4 mm spatial resolution and 5.5 s temporal resolution [39]. DCE MRI data was acquired for 5 min following an injection of 0.1 mmol/kg of Gadobutrol (Gadovist, Bayer Healthcare, Germany). T_1_ maps were estimated by saturation-recovery using 3D GRE images with varying excitation angles. DCE MRI data was analyzed using the extended Tofts model to measure *K^trans^*, *v_e_* and *v_p_*. As shown in figure S2, vascular extravasation fell 88% (p<<0.001) within the tumor ROI. Such a large disruption of the vascular perfusion is not unexpected due to the anti-angiogenic nature of the systemic agent Lenvatinib.


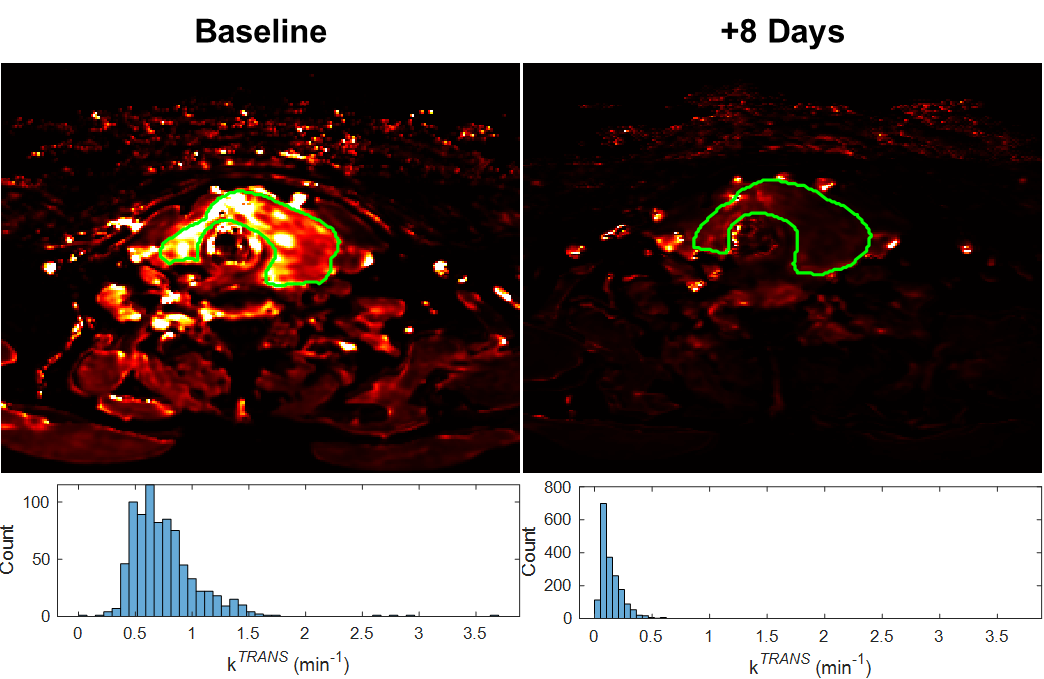


**Figure S2.**  *K^trans^* maps from dynamic contrast enhanced imaging acquired after the hyperpolarized images of a patient with anaplastic thyroid cancer. The *K^trans^* maps prior to therapy show a well perfused thyroid tumor (left). *K^trans^* maps 8 days after initiation of therapy show a poorly perfused tumor (right) indicating a significant vascular response to therapy.

**Supplementary Figure S3**


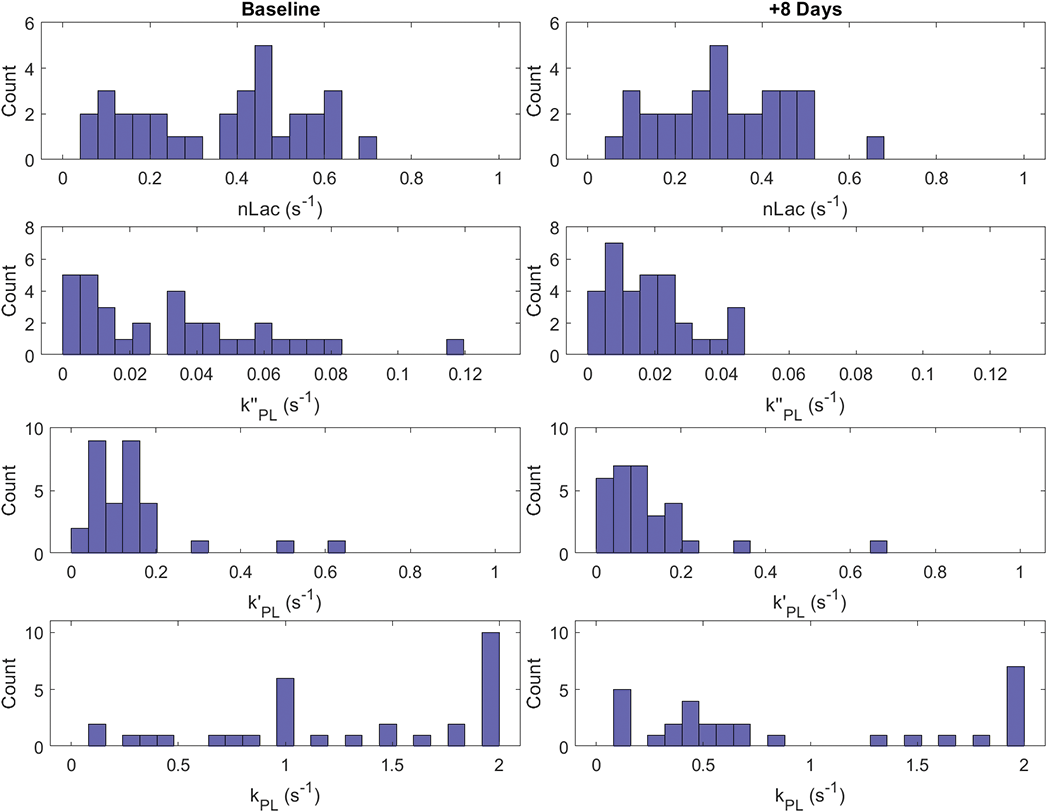


**Figure S3. Histograms of HP MRI metrics in the tumor ROI at baseline and after +8d on therapy.** The normalized lactate ratio (nLac) reflects the area-under-the-curve (AUC) for lactate divided by the sum of AUCs for pyruvate and lactate. $k_{PL}^{''}$ is the apparent rate constant for conversion of HP pyruvate into lactate when quantified using a precursor-product phamarokinetic (PK) model. For a PK model with two physical compartments (vascular, extravascular), $k_{PL}^{'}$ reflects the apparent rate constant for conversion of HP pyruvate into lactate in a well-mixed extravascular environment. In the three-compartment model that accounts for intravascular, extravascular/extracellular, and intracellular space separately, $k_{PL}$ reflects the apparent rate constant for intracellular HP pyruvate metabolism.
